# The Mediator Role of Feelings of Guilt in the Process of Burnout and Psychosomatic Disorders: A Cross-Cultural Study

**DOI:** 10.3389/fpsyg.2021.751211

**Published:** 2021-12-28

**Authors:** Hugo Figueiredo-Ferraz, Pedro R. Gil-Monte, Ester Grau-Alberola, Bruno Ribeiro do Couto

**Affiliations:** ^1^Facultad de Ciencias Sociales y Jurídicas, Universidad Internacional de Valencia, Valencia, Spain; ^2^Department of Social Psychology, Unidad de Investigación Psicosocial de la Conducta Organizacional (UNIPSICO), University of Valencia, Valencia, Spain; ^3^Facultad de Educación, Universidad International de La Rioja (UNIR), Logroño, Spain; ^4^Departamento de Anatomía Humana y Psicobiología, Universidad de Murcia, Murcia, Spain

**Keywords:** burnout, feelings of guilt, psychosomatic disorders, occupational stress, teachers

## Abstract

Burnout was recently declared by WHO as an “occupational phenomenon” in the International Classification of Diseases 11th revision (ICD-11), recognizing burnout as a serious health issue. Earlier studies have shown that feelings of guilt appear to be involved in the burnout process. However, the exact nature of the relationships among burnout, guilt and psychosomatic disorders remains unclear. The purpose of this study was to investigate the mediator role of feelings of guilt in the relationship between burnout and psychosomatic disorders, and perform a cross-cultural validation of the multi-dimensional model by Gil-Monte in two samples of teachers (Portuguese vs. Spanish). The study sample was composed of 1,266 teachers, 1,062 from Spain, and 204 from Portugal. Burnout was measured by the Spanish Burnout Inventory. Hypotheses were tested together in a path model. The results obtained provide empirical evidence for the mediator role of guilt in the relationship between the Burnout syndrome and psychosomatic disorders in the sample of teachers from Spain and Portugal, and they contribute to the empirical validation of the model by Gil-Monte. The results indicate that guilt should be incorporated as a symptom of burnout in order to identify individuals affected by burnout and profiles or types of burnout to differentiate it from other pathologies like depression.

## Introduction

Burnout is a psychological response to chronic work-related stress. Edelwich and Brodsky (1980) have defined burnout as “a progressive loss of idealism, energy, and purpose experienced by people in the helping professions as a result of the conditions of their work.” It is a process that progresses from enthusiasm to stagnation, frustration and, finally, apathy. Price and Murphy (1984) defined burnout as a disordered or unsuccessful process of adaptation to a stressful work situation that progresses from shock and disorganization to volatile emotions (e.g., irony), guilt and loneliness. However, the definition of burnout that is more accepted is the one advanced by Maslach and Jackson (1981), who defined burnout symptoms as: (a) reduced efficacy or personal accomplishment, (b) emotional exhaustion, and (c) depersonalization (or cynicism).

Recently burnout was included in the 11th Revision of the International Classification of Diseases (ICD-11) as an occupational phenomenon (World Health Organization [WHO], 2018).

The research has identified physiological and psychological symptoms associated with burnout among teachers, as cortisol dysregulation (Katz et al., 2016), depressed affect (Szigeti et al., 2017; Martínez et al., 2020), a more negative perception of the general state of health (Simbula, 2010), and cognitive failures that lead to increased distraction, poor performance and inhibition errors (van der Linden et al., 2005), thus affecting the learning environment and interfering with the achievement of educational goals.

Taking into consideration the literature on burnout, it is necessary to continue studying the structure of this phenomenon and the processes underlying the concept of burnout (Cox et al., 2005). Process models on the relationships among the dimensions or symptoms of burnout are confusing and a handicap to the early recognition of burnout. Another gap in the literature is the scarcity of cross-cultural studies (Pines, 2004; Lucchetti et al., 2017).

According to Hofstede and McCrae (2004), culture is the collective programming of the mind that distinguishes one group or category of people from another. The authors highlight that culture is a collective attribute that is not directly visible; it is manifested by behaviors and shared by some people, but not all. For Moreno et al. (2003), cross-cultural studies are essential in psychology, given that they have shown the importance of cultural factors in social and psychological processes. According to Gil-Monte (2008b), there is a danger that theories accepted by part of the world will become universal, leading to their erroneous use in other parts of the globe. Studies on burnout are not exempt from cultural biases.

Although the model of the Maslach Burnout Inventory (MBI; Maslach and Jackson, 1981) has been the dominant paradigm in research on the processes underlying burnout, some alternative models (AMs) have hypothesized different types of burnout that coincide more closely with the clinical experience (Vanheule et al., 2003). In some of these models, feelings of guilt have been identified as one of the most destructive factors in staff burnout (Price and Murphy, 1984). Farber and Miller (1981) identified feelings of guilt as a symptom of teacher burnout. Gil-Monte (2005, 2012) integrated feelings of guilt into a theoretical model to explain different profiles of burnout, in order to reach a more complete diagnosis, identify individuals affected by the syndrome, and recognize the syndrome’s influence on health-related disorders.

Feelings of guilt have also been identified as a symptom of depression. Some studies have concluded that burnout and depression are not separate entities (Bianchi and Brisson, 2017). However, some individuals who display severe burnout also present a depressive disorder (Bianchi et al., 2013). Depressive symptoms, such as dysfunctional attitudes, ruminative responses, pessimistic attributions (Bianchi and Schonfeld, 2016), and attentional and behavioral alterations (Bianchi and Laurent, 2015), have also been observed in individuals with burnout. Consequently, Bianchi et al. (2013) concluded that burnout could be considered equivalent to depressive symptoms in work life. Burnout and depressive symptoms have been found to be inseparably linked, increasing or decreasing together over time (Bianchi et al., 2015).

Nevertheless, the distinction between burnout and depression is partly supported by empirical research (Bianchi and Schonfeld, 2018). Although burnout and depression are positively correlated, they seem to be two distinct constructs (Leiter and Durup, 1994), and distinct phenomena (Toker and Biron, 2012), with job burnout representing a separate diagnostic entity rather than a form of depression (Tement et al., 2016; Parker and Tavella, 2021). Burnout may be a phase in the development of work-related depression, it can co-occur with anxiety and, in the later stages, be accompanied by depressive symptoms (Melamed et al., 2006).

Conclusions about burnout as a form of depression rather than as a differentiated type of pathology have mainly been reached by evaluating burnout levels with the MBI (e.g., Bianchi et al., 2013; Ahola et al., 2014) or the Burnout Measure (BM; Shirom and Ezrachi, 2003). However, as noted by Schaufeli (2003), “the MBI is neither grounded in firm clinical observation nor based on sound theorizing,” and “the BM is inadequate for differentiating burnout from the related but distinct affective states of anxiety and depression” (Shirom and Ezrachi, 2003). Recent studies suggest that the burnout–depression relation is not static but relates to sample and methodological characteristics of examined studies (Meier and Kim, 2021). Koutsimani et al. (2019) indicate that they are different and robust constructs, in this sense, no conclusive overlap between burnout and depression and burnout and anxiety are found.

In addition, different studies have recommended differentiating profiles (Gil-Monte, 2012), types (Farber, 2000), or groups (van Dam, 2016) of burnout to carry out an adequate diagnosis and identify differences between burnout and other clinical disorders such as depression. Gil-Monte, after conducting out clinical interviews to diagnose burnout in nursing professionals (Gil-Monte, 2005) and teachers (Gil-Monte, 2008a), concluded that feelings of guilt is a variable that influences the chronicity of the burnout process, and he differentiates between two profiles of burnout: Profile 1 is less severe, with low levels of guilt, and Profile 2 is more severe, with high levels of guilt.

The aim of the present research is to analyze the mediator role of feelings of guilt in the process of burnout and the relationship with psychosomatic disorders, according to the Gil-Monte (2012) model, and test the model’s invariance in samples of teachers from two countries, i.e., Spain and Portugal. In addition, we investigate the roles of both work overload and autonomy as relevant variables to explain teacher burnout. The findings could contribute to understanding the processes underlying this phenomenon, improving early recognition and to helping to prevent burnout.

The following sections outline teacher burnout and the relationships between work overload, autonomy and the burnout dimensions. In addition, the literature on feelings of guilt and burnout is reviewed, and the role of feelings of guilt in the process of burnout is described according to the model by Gil-Monte (2005). Based on this information, the research hypotheses and the path analysis model are then presented.

Review studies on burnout carried out with teachers suggest that burnout levels among these professionals are high and may be positively associated with poor health (García-Carmona et al., 2019). The research has identified physiological and psychological symptoms associated with burnout among teachers, such as a more negative perception of one’s general state of health and psychosomatic disorders.

Previous studies have identified work overload and autonomy as relevant variables to explain teacher burnout (Alarcon, 2011). According to the Job Strain Model by Karasek (1979), psychological strain results from the interaction between job demands, i.e., work overload- and job decision latitude, i.e., job decision latitude or autonomy. Psychological strain occurs when job demands are high and job decision latitude is low. In a similar vein, the Job Demand-Resource Model of burnout proposes that the interaction between job demands (e.g., work overload) and job resources (e.g., autonomy) is important for the development of job burnout (Demerouti et al., 2001; Demerouti and Bakker, 2011; Bakker and de Vries, 2020).

### Work Overload and Burnout

Work overload is present when the task demands exceed the worker’s capacity to carry them out, that is, when there is an imbalance between what is asked of the worker and what he/she can fulfill, e.g., having large amounts of work, having to work fast, or working under time pressure. Work overload is defined in terms of misfit between the person and the environment. This consideration stipulates that a misfit between demands and abilities in itself does not necessarily constitute overload. Instead, excessive demands produce stress only if the demands have been internalized as goals of the teacher, as when role expectations are accepted by professionals as guidelines for their own behavior. Work overload can be quantitative or qualitative and it has been pointed out in different studies as one of the most intense sources of stress in the professional collective of teachers (Kerr et al., 2011). Various studies with teachers have concluded that work overload is one of the most important predictors of burnout in this collective and strongly related to the emotional component of the syndrome (Salmela-Aro et al., 2019).

### Autonomy and Burnout

Resources in the work environment, such as autonomy, can improve personal growth and the fulfillment of objectives (Demerouti et al., 2001). Many studies have shown that autonomy allows employees to cope with job demands (van der Doef and Maes, 1999), and that it also has motivational potential (Hackman and Oldham, 1980). Recent studies confirm that autonomy might buffer the development of burnout in teachers (Skaalvik and Skaalvik, 2017, 2020).

### Feelings of Guilt and Burnout

Guilt is conceptualized as the unpleasant and remorseful feelings associated with the recognition that one has violated, or is capable of violating, a moral standard. In contrast to shame, wherein the focus of attention is a negative evaluation of the global self, guilt involves a negative evaluation of a specific behavior (Tangney and Tracy, 2012). From an interpersonal approach (Baumeister et al., 1994), guilt is described as a social emotion linked to communal relationships. It is an emotion deeply rooted in two basic affective reactions: empathic activation and anxiety in the face of rejection by others. The origins, functions and process of guilt have important interpersonal aspects, as it is a variable that reinforces ties in relationships. Guilt has the symbolic role of reaffirming commitment toward the other person and the responsibility of taking care of him or her. Feelings of guilt have prosocial effects, as they motivate people to make amends to others, correct their errors and apologize. These interpersonal actions reduce feelings of guilt, and make it possible to alleviate the distress produced by a lack of balance in emotional states resulting from social exchanges (Baumeister et al., 1994). Friberg (2009) states that feelings of guilt would put pressure on an individual that can be reduced through his or her work by helping others.

Although guilt has pro-social effects, because it motivates people to make amends to others (Cohen et al., 2013), excessive or inappropriate levels of guilt can produce a dysfunctional and disruptive experience, as well as psychological and somatization symptoms in some cases (Pineles et al., 2006). Different studies have obtained positive and significant relationships between guilt, anxiety and somatization (Pineles et al., 2006; Cândea and Szentagotai-Tǎta, 2018), and physical and mental health disorders (Spillers et al., 2008; Luck and Luck-Sikorski, 2020). Quiles and Bybee (1997) obtained positive and significant correlations between guilt and anxiety and somatization, and they concluded that chronic feelings of guilt could be an indicator of the use of inappropriate coping strategies and the failure of the individual to regulate his/her emotions. Ghatavi et al. (2002) found that individuals with depression problems had a history of feelings of guilt, and in their conclusions they suggest that guilt may be a variable that predisposes individuals to illness.

Guilt appears to be involved in the burnout syndrome (Farber and Miller, 1981; Maslach, 1982; Price and Murphy, 1984). According to Farber and Miller (1981) “the symptomatic manifestations of teacher burnout are anger, anxiety, irritability (…) cynicism, guilt…” One of the frequent causes of feelings of guilt in professionals is the existence of negative thoughts about others and the negative and cynical way they have treated them. Some professionals underestimate the influence of situations on behavior, and interpret their experiences as reflecting some personality malfunction, leading them to blame themselves for not performing their job adequately. As a result, they develop a sense of failure and loss of self-esteem (Maslach, 1982, pp. 5, 10–12).

On the other hand, these professionals could feel they are becoming cold and dehumanized, and this experience leads them to reaffirm their commitment toward other people and the responsibility of taking care of them (Baumeister et al., 1994), and feel higher levels of burnout. Chang (2009) considers that for many teachers guilt is an unpleasant emotion caused by emotional labor and poor relationships with the students.

Several studies on the process of burnout have included feelings of guilt as a stage in the development of this process. Price and Murphy (1984) state that “a typical burnout victim is a professional full of idealism and a sense of mission.” Staff burnout would progress in seven phases: shock, disorganization, volatile emotions, guilt, loneliness, relief, and re-establishment. These authors consider that feelings of “guilt are among the most destructive factors in the stress syndrome that so often, and so wastefully, results in staff burnout.” According to Burisch (2006, pp. 25–27), burnout would progress in seven stages, and due to low engagement (stage 2), some individuals could develop emotional reactions such as feelings of guilt (stage 3).

In addition, feelings of guilt could explain different types of burnout, considering the role of guilt feelings in the relationship between burnout and its consequences, such as psychosomatic disorders. For example, Vanheule et al. (2003) differentiated two types of high burnout scorers (Type 1 vs. Type 2) taking into consideration how the professionals feel it is their responsibility to fulfill other people’s needs and desires. In this line, Tops et al. (2007) distinguished between burnout individuals with high basal prolactin levels -prolactin profile- vs. low basal prolactin levels. According to the study results, the low prolactin burnout participants scored higher on state negative affect measures, suggesting an important role of decreased dopaminergic functioning in burnout. This role is similar to the relationship between depression and dopamine deficiency (Bloomfield et al., 2019). Contemporary burnout theories have stated that physiological changes in the dopaminergic/motivational system due to overriding fatigue for prolonged periods of time may be fundamental in disorders like burnout (Boksem and Tops, 2008).

### A Model of Burnout

Burnout can be defined as a psychological response to chronic work-related stress that appears in professionals in service organizations who work in direct contact with the clients or users of the organization. It is a non-psychiatric syndrome characterized by cognitive deterioration (i.e., loss of enthusiasm toward the job), emotional deterioration (i.e., psychological exhaustion), and attitudes and behaviors of indifference, withdrawal and, sometimes, abusive attitudes toward the client (i.e., indolence). In addition, in some cases, negative attitudes on the job, especially toward the people with whom the worker establishes work relationships, lead to high feelings of guilt (Gil-Monte, 2005).

Enthusiasm toward the job is a cognitive variable defined as the individual’s desire to achieve goals at work as a source of personal pleasure. This variable is similar to the Personal accomplishment subscale from the MBI (Maslach and Jackson, 1981), but it does not include an indicator of self-efficacy. Psychological exhaustion is defined as the appearance of emotional and physical exhaustion due to the fact that the individual must deal daily with people at work who present problems. This variable is similar to the Exhaustion subscale from the MBI (Maslach and Jackson, 1981), but it includes an aspect of physical exhaustion (i.e., *I feel physically tired at work*). Indolence is the appearance of negative attitudes of indifference and cynicism toward the organization’s clients, such as students and relatives (Richmond et al., 2009, pp. 149–150). This symptom is similar to the Depersonalization subscale from the MBI (Maslach and Jackson, 1981). Guilt is conceptualized as the appearance of feelings of guilt about negative attitudes developed on the job, especially toward the people with whom the individual establishes work relationships. These four symptoms have been brought into a process model on the relationships among the dimensions of burnout (Gil-Monte, 2005, 2012). The “Spanish Burnout Inventory” (SBI; Gil-Monte et al., 2010) was developed to assess these four aspects of burnout. Previous studies have obtained appropriate values of concurrent validity between the SBI and the MBI (Figueiredo-Ferraz et al., 2013).

Gil-Monte (2005) concluded that burnout progresses in a parallel way from low Enthusiasm toward the job and Psychological exhaustion to Indolence. The model states that the burnout process can be understood in terms of the stress-strain-coping framework. Indolence is considered as a dysfunctional, rather than effective, coping strategy that is tried after the reappraisal stage. This approach takes into consideration the model of attitudes and attitude change developed by Eagly and Chaiken (1993), and it integrates the role of cognitive, i.e., Enthusiasm toward the job – and emotional, i.e., Psychological exhaustion – experiences as mediators in the relationship between perceived job stress and behavioral/attitudinal outcomes.

**FIGURE 1:**
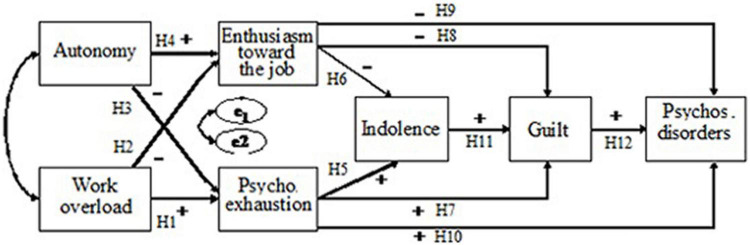
Hypothesized model.

Taking this into account, Gil-Monte (2005) proposes two profiles of workers in the process of developing burnout (see Figure 1). On the one hand, we have the workers for whom strategies of distancing are helpful in order to be able to face disillusionment with work and psychological deterioration. These strategies often include laziness and cynical behavior toward customers, leading to deterioration in the quality of the service provided and causing complaints from customers. However, these workers are usually comfortable with this situation, because it means that they can remain in their jobs for many years, without suffering any real consequences to their health. This profile is called Profile 1.

On the other hand, Profile 2 indicates workers for whom these coping strategies have not proved to be effective in handling problems. These workers feel guilty for the treatment they give to the clients of the organization. As noted above, guilt has a high interpersonal component. In these cases, workers feel that they are violating the ethical norms of the organization, often leading them to develop negative consequences to the point where they acquire medical leave rather than go to work. These consequences, derived from guilt, as presented above, can cause more severe manifestations of burnout, and health-related disorders (Guidetti et al., 2017) such as anxiety, psychosomatic problems and depression (Gil-Monte, 2012) and inclination toward absenteeism (Rabasa et al., 2016).

### The Present Study

The aim of this study was to investigate the mediator role of guilt feelings in the relationship between burnout and psychosomatic disorders, based on the Gil-Monte (2012) burnout model, and analyze the cross-national invariance, i.e., Spanish sample vs. Portuguese sample- of the hypothesized model. Based on previous research, we hypothesized twelve relationships among the variables included in the study. These hypotheses (H1 to H12) were tested together in a path model to determine the mediator role of guilt -as a symptom of burnout- in the development of the burnout process, and its relationship with levels of psychosomatic disorders (Figure 1).

To test the cross-national validity of the model, Hypothesis 13 was formulated: There will be no significant differences in the degree of fit of the path analysis models, or in the size of the relationships among the variables in the model, based on the nationality of the sample (Portugal vs. Spain).

## Materials and Methods

### Participants

The total sample in the study was composed of 1,266 participants. As this study has a cross-cultural nature, the description of the sample will be carried out separately by country.

The Portuguese sample consisted of 204 teachers. In all, 250 questionnaires were distributed non-randomly in different schools in northern Portugal. The response rate in the study was 81.8%. Regarding the sociodemographic variables considered for the study, the composition was the following. In terms of sex, 59 participants were men (29%), and 145 women (71%). Regarding the number of years in the profession, the mean was 17.5 (*sd* = 9.7, max. = 37, min. = 0). The mean age of the sample was 41.3 (*sd* = 9.2, max. = 68, min. = 23).

The Spanish sample was composed of 1,062 teachers. In all, 1,600 questionnaires were distributed non-randomly in different schools. The study response rate was 66.38%. Regarding the sociodemographic variables considered for the study, the composition is the following. In terms of sex, 294 participants were men (27.7%), 762 women (71.8%), and 6 participants did not answer this question (0.6%). Regarding the number of years in the profession, the mean was 18.1 (*sd* = 10.2, max. = 46, min. = 0). The mean age of the sample was 41.7 (*sd* = 9.6, max. = 68, min. = 21).

### Instruments

Work overload was evaluated with the Work overload subscale from the UNIPSICO questionnaire (Gil-Monte, 2012, 2016a), composed of 6 items (Portugal: α = 0.67; Spain: α = 0.72; e.g., “*When you are working, do you encounter especially difficult situations?*”). Autonomy (5 items; Portugal: α = 0.74; Spain: α = 0.83) was evaluated with the Autonomy Scale from the UNIPSICO questionnaire (Gil-Monte, 2016b). The items refer to the discretion given to the worker in managing his/her work and rest time. The employee is asked about the choice of work rhythm, the freedom to alter it if he/she so desires, independence in making decisions, etc. (e.g., “*In this job, I have the freedom to decide how to do it*”).

Burnout was measured by the SBI (Gil-Monte, 2011; Figueiredo-Ferraz et al., 2013; Olivares et al., 2018). This instrument contains 20 items distributed into four dimensions called: (1) Enthusiasm toward the job: the individual’s desire to achieve goals at work because it is a source of personal pleasure (5 items, Portugal: α = 0.87; Spain: α = 0.89); (2) Psychological exhaustion: the appearance of emotional and physical exhaustion due to the fact that he or she must deal daily with people at work who present problems (4 items, Portugal: α = 0.82; Spain: α = 0.85); (3) Indolence: the appearance of negative attitudes of indifference and cynicism toward the organization’s clients (6 items, Portugal: α = 0.75; Spain: α = 0.72); and (4) Guilt: the appearance of feelings of guilt about negative attitudes developed on the job, especially toward the people with whom he or she establishes work relationships (5 items, Portugal: α = 0.76; Spain: α = 0.83). Low scores on Enthusiasm toward the job, together with high scores on Psychological Exhaustion, Indolence, and Guilt, indicate high levels of burnout.

Psychosomatic disorders were measured by the UNIPSICO subscale (9 items, Portugal: α = 0.90; Spain: α = 0.86; Gil-Monte, 2016a). Items include different work-related psychosomatic disorders (e.g., headaches, musculoskeletal pain, sleep quality, anxiety, and sickness; e.g., “*Do you have a headache?*”). Participants answered the items on all scales on a 5-point frequency scale ranging from “Never” (0) to “Very frequently: Every day” (4).

### Procedure

For data collection, contact was first made with the administration of the teaching institutions, and the aim of the study was presented in order to obtain authorization and support for applying the instruments. The instruments were handed to the teachers personally. Teachers and principals of the teaching institutions were informed about the research, which would not have any individual and/or institutional assessment effects, and the answers would be anonymous and confidential. The participants were selected in a non-random manner, and the participation was voluntary. The questionnaire was self-administered; it was handed to teachers at the beginning of their working day and they were asked to return the completed questionnaire at the end of the day by dropping it in a box in the teachers’ room. The study was approved by the Ethics Committee of the University of Valencia, Spain.

### Data Analysis

To test the model, structural equation analysis was performed with the AMOS 25.0 program. Taking into consideration the moderate size of the Portuguese sample, path analysis, rather than SEM, was used to test the hypothesized model. The Maximum likelihood estimation method was used to test the model. Various indices are suggested to test the model’s fit, such as the χ^2^ statistic and associated probability level. Because of the sensitivity of the statistic χ^2^ to the sample size, we propose other fit indices. The *Goodness of Fit Index* (GFI) is a measure of the relative amount of variance and covariance explained by the model. The *Adjusted Goodness of Fit Index* (AGFI) adjusts the GFI based upon degrees of freedom. The *Normed Fit Index* (NFI) and the *Comparative Fit Index* (CFI) indicate the amount of variation and covariation accounted for by a particular model by comparing the relative fit of the given model with the fit of a baseline model. Some authors have recommended at least values above 0.90 for these indices as indicators of a good fit of the model (Bentler, 1992; Hoyle, 1995). The *Root Mean Square Error of Approximation* (RMSEA) estimates the overall error amount in the model. Values between 0.05 and 0.08 indicate an adequate fit of the model (Hu and Bentler, 1999).

Bootstrapping, with the number of bootstrap samples set at 5,000, was carried out to estimate 95% confidence intervals. Preacher and Hayes (2008) have recommended bootstrapping for testing mediation, as it does not require normality of the sampling distribution of the indirect effects.

## Results

Table 1 shows the means, standard deviations, range, skewness, kurtosis, and internal consistencies of all the scales included in this study. All scales showed good reliabilities, with Cronbach’s alpha coefficients. Table 2 shows the correlations between all the scales included in this study.

**TABLE 1:** Means, standard deviations, range, skewness, kurtosis and internal consistencies (Cronbach’s alphas), for the two study samples.

	Mean		*Sd*		Range	Kurtosis		Skewness		Alpha	
	Sp	Pt	Sp	Pt		Sp	Pt	Sp	Pt	Sp	Pt
1. Work overload	1.79	1.86	0.67	0.60	0–4	−0.27	0.22	0.16	0.24	0.72	0.67
2. Autonomy	2.57	2.73	0.59	0.62	0–4	−0.09	−0.36	−0.43	−0.12	0.83	0.74
3. Enthusiasm toward the job	2.97	2.87	0.72	0.70	0–4	0.42	−0.62	−0.66	−0.30	0.87	0.87
4. Psychological exhaustion	1.87	1.87	0.84	0.84	0–4	−0.30	−0.29	0.21	0.24	0.82	0.82
5. Indolence	1.20	1.22	0.60	0.62	0–4	0.28	−0.19	0.55	0.39	0.75	0.75
6. Guilt	0.96	0.94	0.63	0.60	0–4	0.53	−0.26	0.62	0.44	0.76	0.76
7. Psychosomatic disorders	1.14	0.98	0.73	0.82	0–4	0.29	−0.05	0.74	0.78	0.90	0.90

*Sp, Spanish sample; Pt, Portuguese sample.*

**TABLE 2:** Correlations between variables of the study.

	1	2	3	4	5	6	7
1. Work overload	1	−0.17*	−0.29**	0.47**	0.34**	0.35**	0.36**
2. Autonomy	−0.26**	1	0.29**	−0.24**	−0.14*	−0.20**	−0.14
3. Enthusiasm toward the job	−0.23**	0.41**	1	−0.42**	−0.35**	−0.15*	−0.17*
4. Psychological exhaustion	0.59**	−0.22**	−0.33**	1	0.42**	0.36**	0.47**
5. Indolence	0.27**	−0.07*	−0.31**	0.35**	1	0.41**	0.23**
6. Guilt	0.28**	−0.07*	−0.19**	0.31**	0.43**	1	0.29**
7. Psychosomatic disorders	0.43**	−0.19**	−0.21**	0.51**	0.19**	0.21**	1

**p < 0.01, *p < 0.05.

*The correlations for the Spanish sample are in the lower part of the diagonal, and those for the Portuguese sample are in the upper part.*

The Hypothesized model yielded a significant χ^2^ value for the two samples: Portugal [χ^2^_(7)_ = 19.13, *p* < 0.05] and Spain [χ^2^_(7)_ = 72.90, *p* < 0.05], which indicates an insufficient model fit. However, some of the values obtained for other fit indices were acceptable for the two samples: GFI = 0.975, AGFI = 0.900, NFI = 0.932, CFI = 0.954 for the Portuguese sample; and GFI = 0.981, AGFI = 0.925, NFI = 0.957, CFI = 0.960 for the Spanish sample. The values obtained in the Spanish sample for RMSEA = 0.094 and for the Portuguese sample, RMSEA = 0.092, indicate that the hypothesized model presented a mediocre fit in the two study samples.

A review of the parameter estimations for the relationships indicated that two relations in the hypothesized model were not significant in either sample: the relation between Enthusiasm toward the job and Guilt (Spain, β = −0.01, *p* = 0.590; Portugal, β = −0.06, *p* = 0.340; Hypothesis 8); and the relation between Enthusiasm toward the job and Psychosomatic problems (Spain, β = −0.04, *p* = 0.130; Portugal, β = −0.02, *p* = 0.030; Hypothesis 9).

**FIGURE 2:**
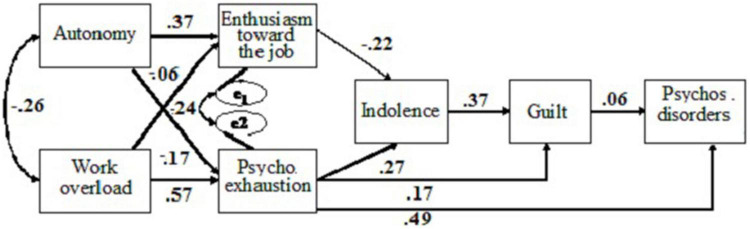
Standardized coefficients for the modified model, taking into consideration feelings of guilt as predictor of psychosomatic disorders for the Spanish sample.

**FIGURE 3:**
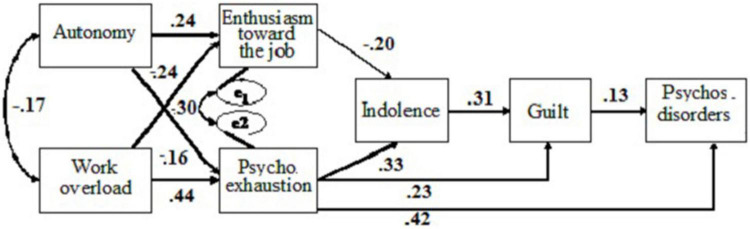
Standardized coefficients for the modified model, taking into consideration feelings of guilt as predictor of psychosomatic disorders for the Portuguese sample.

Considering these results, an alternative model (AM) was evaluated, eliminating the non-significant relationships in both samples. The modified model yielded a significant χ^2^ value for the two samples: Portugal, χ^2^_(9)_ = 20.20, *p* < 0.05; and Spain, χ^2^_(9)_ = 75.46, *p* < 0.05, which indicates an insufficient model fit. However, for the two samples, it showed a good fit to data according to: GFI = 0.973, AGFI = 0.917, NFI = 0.929, CFI = 0.957 for the Portuguese sample, and GFI = 0.981, AGFI = 0.940, NFI = 0.954, CFI = 0.959 for the Spanish sample. The fit to data was adequate according to RMSEA = 0.078 (Portugal), 0.083 (Spain). All the hypothesized relationships in the revised model were confirmed (Hypothesis 1 to Hypothesis 7; and Hypothesis 10 to Hypothesis 12; Figure 2 for the Spanish sample and Figure 3 for the Portuguese sample).

**FIGURE 4:**
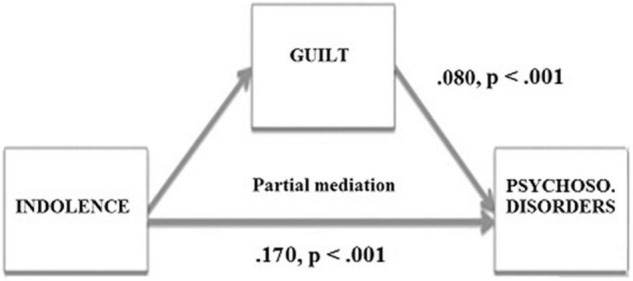
Mediation of the variable Guilt in the relationship between the SQT and Psychosomatic disorders in the Spanish sample.

In the Spanish sample, based on bootstrapping the standardized indirect effect of Indolence on Psychosomatic disorders was 0.08 (*p* < 0.001; bias corrected 95% CI:0.044 to 0.122). In line with Hypothesis 11 and Hypothesis 12, the relationship between Indolence and Psychosomatic disorders was mediated by feelings of guilt. However, the direct effect of Indolence on Psychosomatic disorders was significant (0.170, *p* < 0.001; bias corrected 95% CI:0.073 to 0.240), which indicates a partial mediation effect of the Guilt variable (Figure 4).

**FIGURE 5:**
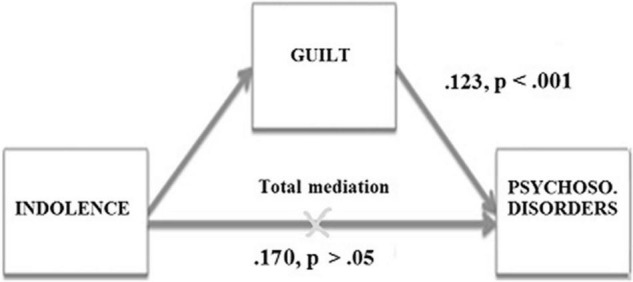
Mediation of the variable Guilt in the relationship between the SQT and Psychosomatic disorders in the Portuguese sample.

In the Portuguese sample, the standardized indirect effect of Indolence on Psychosomatic disorders was 0.123 (*p* < 0.001; bias corrected 95% CI:0.056 to 0.214). In line with Hypothesis 11 and Hypothesis 12, the relationship between Indolence and Psychosomatic disorders was mediated by feelings of guilt. Moreover, the direct effect of Indolence on Psychosomatic disorders was no longer significant (0.170, *p* > 0.05 bias corrected 95% CI:0.000 to 0.359), which indicates a total mediation effect of the Guilt variable (Figure 5).

To test Hypothesis 13, an analysis of invariance was performed. The invariance of the AM was shown in both samples, adding a series of restrictions on some parameters (Byrne, 1998). All of the relations between variables and the covariances between the errors were balanced in the two samples. In this case, the unrestricted model obtained a value χ^2^_(18)_ = 95.677 and the completely restricted model χ^2^_(25)_ = 109.319, with a difference of χ^2^_(7)_ = 13.642, *p* > 0.05. The model was invariant regardless of the origin of the sample (Spanish vs. Portuguese).

Differences in the relations between the variables in the two models were analyzed to find out whether there were significant differences according to where the sample was from Table 3. No significant differences were found in the relations that explain the burnout development process based on the origin of the sample. This result indicates that the model is invariant for all the hypothesized relationships in the AM.

**TABLE 3:** Parameters for the relationships among the variables, *Z* value for the differences and significance level.

			Spanish sample		Portuguese sample		
			Values	*p*	Values	*p*	*Z* value
Autonomy	→	Enthusiasm toward job	γ = 0.449	0.000	γ = 0.275	0.000	2.61**
Work overload	→	Psychological exhaustion	γ = 0.715	0.000	γ = 0.620	0.000	2.24*
Work overload	→	Enthusiasm toward job	γ = −0.147	0.000	γ = −0.286	0.000	1.90
Autonomy	→	Psychological exhaustion	γ = −0.089	0.014	γ = −0.221	0.008	1.76
Psychological exhaustion	→	Indolence	β = 0.197	0.000	β = 0.248	0.000	−0.70
Enthusiasm toward job	→	Indolence	β = −0.182	0.000	β = −0.181	0.003	−0.01
Indolence	→	Guilt	β = 0.392	0.000	β = 0.301	0.000	1.35
Psychological exhaustion	→	Guilt	β = 0.130	0.000	β = 0.168	0.000	−0,51
Guilt	→	Psychosomatic disorders	β = 0.073	0.024	β = 0.174	0.050	-1.33
Psychological exhaustion	→	Psychosomatic disorders	β = 0.426	0.000	β = 0.410	0.000	0.25

**p < 0.01 and *p < 0.05.

In addition, because sex is a sociodemographic variable that explains significant differences in burnout (Purvanova and Muros, 2010), and because the number of women in both samples was higher than the number of men, the sex invariance of the AM was tested. The model yielded a significant χ^2^ value for the two samples: Women, χ^2^_(9)_ = 78.02, *p* < 0.05; and men, χ^2^_(9)_ = 19.68, *p* < 0.05, which indicates an insufficient model fit. However, for the two samples, it showed a good fit to data according to: GFI = 0.977, AGFI = 0.927, NFI = 0.945, CFI = 0.951 for the women’s sample, and GFI = 0.984, AGFI = 0.952, NFI = 0.960, CFI = 0.977 for the men’s sample. The fit to data was adequate according to RMSEA = 0.092 (women) and 0.058 (men). All the hypothesized relationships in the revised model were confirmed, with the exception of H2 (Work overload-Enthusiasm toward the job relationship, β = −0.04, *p* = 0.429) and H3 (Autonomy-Psychological exhaustion relationship, β = −0.08, *p* = 0.073) in the men’s sample.

An analysis of invariance was performed. The unrestricted model obtained a value χ^2^_(18)_ = 97.682, and the completely restricted model χ^2^_(25)_ = 120.808, with a difference of χ^2^_(7)_ = 23.126, *p* < 0.01. The model was not invariant, regardless of the sex of the participants. Significant differences were found in the relations between Work overload – Enthusiasm toward the job (*z* = −2.99, *p* < 0.001; β = −0.23, *p* < 0.001 in the women’s sample, and β = −0.04, *p* > 0.05 in the men’s sample), Guilt – Psychosomatic disorders (*z* = −1.90, *p* < 0.05; β = 0.08, *p* < 0.05 in the women’s sample, and β = 0.19, *p* < 0.001 in the men’s sample), and Psychological exhaustion – Psychosomatic disorders (*z* = 3.46, *p* < 0.001; β = 0.50, *p* < 0.001 in the women’s sample, and β = 0.29, *p* < 0.001 in the men’s sample).

In the women’s sample, based on bootstrapping, the standardized indirect effect of Indolence on Psychosomatic disorders was 0.07 (*p* < 0.001; bias corrected 95% CI:0.039 to 0.094). However, the direct effect of Indolence on Psychosomatic disorders was significant (0.138, *p* < 0.001; bias corrected 95% CI:0.076 to 0.198), which indicates a partial mediation effect of the Guilt variable. In the men’s sample, similar results were obtained. The standardized indirect effect of Indolence on Psychosomatic disorders was 0.11 (*p* < 0.001; bias corrected 95% CI:0.061 to 0.157); and the direct effect of Indolence on Psychosomatic disorders was significant (0.157, *p* < 0.001; bias corrected 95% CI:0.070 to 0.242), which indicates a partial mediation effect of the Guilt variable.

## Discussion

The purpose of this study was to analyze the mediator role of feelings of guilt in the relationship between burnout and psychosomatic disorders and test the invariance of the model based on the origin of the sample (Spain vs. Portugal). The results indicate that the hypothesized model (i.e., Indolence → Guilt →Psychosomatic disorders) is a good representation of the burnout process and its relationship with psychosomatic disorders, and they provide support for the mediator role of feelings of guilt in the relationship between burnout (i.e., levels of indolence) and psychosomatic disorders. As zero was not in the confidence interval (CI:0.044 to 0.122) for the Spanish sample or the Portuguese sample (CI:0.056 to 0.214), we can conclude that the indirect effect is different from zero. Therefore, in our study, higher levels of indolence were associated with higher levels of guilt, which were in turn associated with higher levels of psychosomatic disorders. Comparing the model according to the origin of the sample, its invariance is confirmed (i.e., Indolence → Guilt → Psychosomatic disorders), which means the model is a good representation of the development of the burnout syndrome in teachers in these two countries.

Previous studies have obtained construct validity for the SBI in Portuguese (Figueiredo-Ferraz et al., 2013) and Spanish (Gil-Monte, 2011), but until this study was carried out, there were no cross-cultural evaluations of the theoretical model of the burnout process derived from the SBI psychometric model. Culture is an important variable in the evaluation of psychological problems such as burnout syndrome. Wierzbicka (1995) indicates the need to take into account that emotions are specific to each culture, and that some English terms referring to emotions may not have semantic equivalents in other languages, which can lead to evaluating different concepts even when attempting to evaluate the same one. The central concept in this study is guilt and its role in the burnout development process. Guilt is a cultural emotion, and it is often difficult to find equivalent concepts, depending on the geographic zone or the religion. The results of the study suggest that this is not true in the case of the Spanish and Portuguese samples, and for the burnout syndrome development process.

The results obtained present various similarities in the two samples in the study (Spain vs. Portugal). The descriptive analysis shows that the highest mean was obtained for the variable Enthusiasm toward the job, and the lowest mean was found for the variable Guilt in both samples. The analysis of the Pearson r correlations indicates that the most intense correlations were obtained between the same variables in both samples, i.e., Psychological exhaustion – Work overload, Psychological exhaustion – Psychosomatic disorders, and Indolence – Guilt. These similarities are also present in the results of the hypothesized path analysis model. In both samples, 10 of the 12 study hypotheses were confirmed, and in both studies, Hypotheses 8 (Enthusiasm toward the job – Guilt) and 9 (Enthusiasm toward the job – Psychosomatic disorders) were not confirmed. Together, the similarities in the results of both samples for the path analysis model made it possible to confirm study Hypothesis 13, which hypothesized the invariance of the model.

Taking into consideration the gender of the participants in the study, an adequate fit of the model to the data was obtained for the sample of men and for the sample of women. Empirical support was also obtained for the partial mediation of Guilt in the relationship between Indolence and Psychosomatic disorders. However, the model was not invariant when comparing the results for the two samples. The relationship between Work overload and Enthusiasm toward the job, and between Psychological exhaustion and Psychosomatic disorders, was significantly more intense in the sample of women than in the sample of men, whereas the relationship between Guilt and Psychosomatic disorders was significantly more intense in the sample of men than in the sample of women. Coinciding with the results obtained in previous studies (Purvanova and Muros, 2010; Tement et al., 2016; Marchand et al., 2018), our results suggest that it is necessary to consider gender differences when studying the relationships between the predictors of burnout and its symptoms, and between the symptoms and their consequences. However, the burnout syndrome development process, according to the hypothesized model, i.e., the relationship among its symptoms and between the symptoms and health problems- is similar for men and women.

Our results have replicated the results of the Gil-Monte (2012) study, as they show that it seems appropriate to establish a relationship from both enthusiasm toward the job and psychological exhaustion to indolence and from indolence to guilt. In addition, the results contribute to supporting the specification of the burnout process according to the model designed by Gil-Monte (2005), taking into consideration the model of attitudes and attitude change (Eagly and Chaiken, 1993) to explain the relationship among the burnout dimensions (enthusiasm toward to indolence; psychological exhaustion to indolence). The study contributes to understanding the processes underlying the burnout concept (Cox et al., 2005). We can conclude that burnout progresses in a parallel way from enthusiasm toward the job and psychological exhaustion to indolence.

The results of the study support the mediator role of the levels of guilt feelings about negative attitudes and behaviors at work in the relationship between burnout and its health-related consequences. They indicate that guilt feelings contribute to explaining the existence of different forms of the evolution of burnout linked to the development of guilt (Gil-Monte, 2012; Rabasa et al., 2016); thus, different types of burnout (Vanheule et al., 2003; Tops et al., 2007; Boersma and Lindblom, 2009) could be explained by considering the role of guilt feelings in the relationship between burnout and its consequences.

Based on the theoretical model underlying the SBI (Gil-Monte, 2005, 2012), it is possible to distinguish two profiles in the development of burnout. In both profiles, indolence can be understood as a coping strategy that arises to handle the perception of low enthusiasm toward the job and high psychological exhaustion levels. While for some teachers, i.e., Profile 1- this coping strategy is sufficient for them to manage the levels of strain, other teachers, i.e., Profile 2- feel remorse and reaffirm their commitment toward students and relatives as a restorative behavior to alleviate emotional distress. Professionals who develop aggression blame others for difficulties and problems, and resentment toward them is less restrained (Maslach, 1982, p. 12), in contrast to professionals who develop feelings of guilt and depression because they blamed themselves.

The feelings of guilt would put pressure on an individual that can be reduced through his or her work by helping others. Taking this suggestion into consideration, Profile 2, according to the SBI, could explain why in some cases burnout levels are higher in individuals distinguished by high commitment and outstanding performance. Individuals fitting Profile 2 will develop high commitment in their jobs helping others as a way to reduce feelings of guilt (Baumeister et al., 1994) and alleviate the emotional distress resulting from taking responsibility for causing others’ suffering. However, as stressful working conditions do not change, there is a loop over time that produces a dysfunctional and disruptive experience, and later psychosomatic disorders.

According to Chang (2009), guilt appears to contribute to teacher burnout in terms of prolonged stressors, and Quiles and Bybee (1997) concluded that chronic feelings of guilt could be an indicator of the failure of the individual to regulate his/her emotions. In addition, physiological changes related to decreased dopaminergic functioning due to prolonged periods of stress (Bloomfield et al., 2019) could explain Profile 2 and the relationship between excessive feelings of guilt and psychosomatic disorders (Pineles et al., 2006). Different studies have obtained positive and significant relationships between guilt, anxiety and somatization (Cândea and Szentagotai-Tǎta, 2018) and physical and mental health disorders (Spillers et al., 2008), and some of them suggest that guilt could be a variable that predisposes individuals to illness (Ghatavi et al., 2002).

## Limitations and Future Research

This study has some limitations. First, the cross-sectional nature of the data does not provide answers about the direction of causality between guilt and psychosomatic disorders, and mediated effects estimates can be biased. Longitudinal studies are needed to reach conclusions on this issue. Second, the study only focuses on teachers, which restricts the generalizability of the results. Therefore, we suggest that other organizational contexts be studied in future research. Third, the data derived entirely from self-report questionnaires, which increased the likelihood of common method variance effects. We used several methods to minimize the impact of these biases (Podsakoff et al., 2003): (a) participants’ confidentiality was guaranteed; (b) participants did not know which items belonged to which scales; (c) back-translation minimized item ambiguity; (d) taking into consideration the scale length, predictor and criterion variables were included in the questionnaire, together with other scales evaluating different constructs (e.g., work overload, autonomy), and they were placed in different positions in the questionnaire; and (e) Harman’s single factor test was performed in both samples, and the first factor did not account for the majority of the variance in the items (Portuguese sample, 29.18%; Spanish sample, 29.08%), suggesting that common method variance is not of great concern. Fourth, the Spanish sample is larger than the Portuguese sample, and this difference could lead to a statistically significant bias in favor of the larger sample. Five, participant selection was non-random. However, designing a study of these characteristics with a random sample can lead to a very low response rate, and the data collection time would be excessively long, questioning the stability of the environmental conditions at the moment when the sample was gathered.

Future research should continue to investigate the processes through which guilt generates positive effects, and when it does not. Some studies suggest that there are differences in the relationship between chronic vs. pre-dispositional guilt and indices of depression and mental health (Quiles and Bybee, 1997). It would be interesting to analyze which individual and situational factors cause guilt in the process of burnout and burnout Profile 2. In addition to the mediator effects of feelings of guilt, moderator effects of this variable in the relationship between indolence and health complaints should also be tested.

## Conclusion and Practical Implications

This study contributes to understanding the processes underlying the burnout phenomenon (Cox et al., 2005), and it highlights the importance of the role of guilt in the development of burnout in Spanish and Portuguese teachers.

The results of the present study recommend incorporating the evaluation of guilt as a symptom of burnout, in order to reach a more complete diagnosis, discriminate among individuals affected by the syndrome, and recognize the syndrome’s influence on health-related disorders. More recently, according to the existence of the different Burnout profiles, Leiter and Maslach (2016) point out the importance of identifying multiple person-centered profiles along the burnout-commitment continuum.

Moreover, they recommend considering the existence of burnout profiles (Gil-Monte, 2012), types (Farber, 2000), or groups (van Dam, 2016) as a strategy to differentiate burnout from other pathologies, such as depression. Based on the model by Gil-Monte (2005, 2012), it is possible to identify a burnout profile that does not overlap with depression (Profile 1) and a profile that is closer to depression (Profile 2). The development process of both profiles does not match the development of depression (Gil-Monte, 2008a). Thus, it seems more reasonable that, in the later stages of some types of burnout, i.e., Profile 2-, symptoms appear that are also found in other pathologies (Melamed et al., 2006), as in the case of feelings of guilt.

We agree that burnout and depression are distinct health disorders (Toker and Biron, 2012), with job burnout representing a separate diagnostic entity rather than a form of depression (Tement et al., 2016). Although many interesting and rigorous studies have concluded that the current state of science suggests that burnout is a form of depression rather than a differentiated type of pathology (Ahola et al., 2014; Bianchi et al., 2015; Schonfeld and Bianchi, 2016), their conclusions have been drawn without taking the burnout profiles into consideration and using the MBI as the instrument to evaluate burnout. These limitations should be addressed in future studies.

The study may be an important point of reference for clinicians and therapists, facilitating both the diagnosis and treatment of different types of burnout.

Although our study has been carry out in a sample of teachers, burnout also affects members of other helping professions. The health deterioration process described above may experience an increase when people have to take care of someone suffering from a chronic disease, or when professionals have to do their work in extreme situations, such as taking care of victims of natural disasters. The study provides a useful addition to the tools for the diagnosis of burnout. Making diagnoses in the initial stages of burnout could avoid the increase in intensity of the symptoms and make it possible for an earlier recovery.

On the other hand, the keys to intervening in order to reduce work stress and to prevent negative consequences for the teachers and for the organization reside in two fundamental methods: prevention and adequate training (Iancu et al., 2017).

Learning to cope with stress can prevent the occurrence of more serious consequences, such as the development of burnout and acute health problems.

This study could also be useful in detecting the need for intervention programs to eliminate sources of stress and the need to train teachers in techniques for dealing with stress. Regarding results, managers might consider how a person’s proneness to guilt differs depending on contextual sources related to, for example, how well resources are managed, and carry out programs to prevent Profile 2. Alternatively, they can help to promote collaborative relationships for teachers reporting high burnout, increase teacher autonomy, and enhance skills and capacities related to efficacious teaching (Gil-Monte, 2019).

## Data Availability Statement

The raw data supporting the conclusions of this article will be made available by the authors, without undue reservation.

## Ethics Statement

The studies involving human participants were reviewed and approved by University of Valencia. The patients/participants provided their written informed consent to participate in this study.

## Author Contributions

HF-F and PG-M were substantially involved in planning and conducting the study; made a substantial contribution to the concept or design of the work and acquisition, analysis, or interpretation of data; drafted the article or revised it critically for important intellectual content; approved the version to be published; and have participated sufficiently in the work to take public responsibility for appropriate portions of the content. EG-A and BR were substantially involved in conducting the study and made a contribution to analysis or interpretation of data; drafted the article or revised it critically for important intellectual content; approved the version to be published; and have participated sufficiently in the work to take public responsibility for appropriate portions of the content. All authors contributed to the article and approved the submitted version.

## Conflict of Interest

The authors declare that the research was conducted in the absence of any commercial or financial relationships that could be construed as a potential conflict of interest.

## Publisher’s Note

All claims expressed in this article are solely those of the authors and do not necessarily represent those of their affiliated organizations, or those of the publisher, the editors and the reviewers. Any product that may be evaluated in this article, or claim that may be made by its manufacturer, is not guaranteed or endorsed by the publisher.
